# Viral Proteins as Emerging Cancer Therapeutics

**DOI:** 10.3390/cancers13092199

**Published:** 2021-05-03

**Authors:** Ekta Manocha, Arnaldo Caruso, Francesca Caccuri

**Affiliations:** Section of Microbiology, Department of Molecular and Translational Medicine, University of Brescia Medical School, 25123 Brescia, Italy; ekta.manocha@unibs.it (E.M.); arnaldo.caruso@unibs.it (A.C.)

**Keywords:** viral proteins, anticancer drugs, cell cycle, proliferation, apoptosis

## Abstract

**Simple Summary:**

This review is focused on enlisting viral proteins from different host sources, irrespective of their origin, that may act as future cancer curatives. Unlike the viral proteins that are responsible for tumor progression, these newly emerged viral proteins function as tumor suppressors. Their ability to regulate various cell signaling mechanisms specifically in cancer cells makes them interesting candidates to explore their use in cancer therapy. The discussion about such viral components may provide new insights into cancer treatment in the absence of any adverse effects to normal cells. The study also highlights avian viral proteins as a substitute to human oncolytic viruses for their ability to evade pre-existing immunity.

**Abstract:**

Viruses are obligatory intracellular parasites that originated millions of years ago. Viral elements cover almost half of the human genome sequence and have evolved as genetic blueprints in humans. They have existed as endosymbionts as they are largely dependent on host cell metabolism. Viral proteins are known to regulate different mechanisms in the host cells by hijacking cellular metabolism to benefit viral replication. Amicable viral proteins, on the other hand, from several viruses can participate in mediating growth retardation of cancer cells based on genetic abnormalities while sparing normal cells. These proteins exert discreet yet converging pathways to regulate events like cell cycle and apoptosis in human cancer cells. This property of viral proteins could be harnessed for their use in cancer therapy. In this review, we discuss viral proteins from different sources as potential anticancer therapeutics.

## 1. Introduction

Viruses have proven to be drivers of evolution since more than 500 million years ago. They have turned out to be obligatory intracellular parasites and shaped genomes by supplying essential mechanisms. They have adapted not just to eukaryotic cells but also prokaryotes for maintenance of the lysogeny state of phages inside bacteria [1]. DNA viruses have been evolving and diversifying for millions of years while RNA viruses are probably having a more recent evolution and human adaptation for only thousands of years [2]. Both DNA and RNA viruses trigger metabolic reprograming and hijack mechanisms like cell cycle and cell signaling in the host cells to facilitate optimal virus production [3].

Viral genomes consist of two transcriptional units encoding for non-structural and structural proteins. Structural proteins are components of virus particles and perform functions like cell recognition, fusion, entry, or replication [4]. On the other hand, non-structural (NS) proteins have been involved with cellular hijacking mechanisms like inclusions formation, cytoskeleton interaction, apoptosis, and autophagy [5]. Not all NS proteins are known for their functions, but several of them are involved in the virus replication cycle and latency. Many of NS proteins are conserved and share homology with other viruses, e.g., Rep proteins, adeno-associated virus (AAV) Rep78 and human herpesvirus type 6 (HHV-6) Rep6 share salient structural similarities for which they were also considered to share functional similarities [6].

Different viral proteins exert discreet yet converging pathways to regulate events like cell cycle and apoptosis selectively in human cancer cells based on present aberrations. However, the functions and mechanisms behind cancer suppression activity of the viral proteins have not been completely deciphered yet. Nonetheless, enlisted molecules in this review are potential subjects to explore and may pave the way to be novel candidates in anticancer therapy. In this review, we discuss some of the proteins from human viruses widely known for their growth hampering roles in cancer cells followed by a few newly examined proteins from avian and alphaviruses, their mechanisms of action, role in cell cycle, and unknowns to be elucidated in future studies.

## 2. Human Viral Proteins as Anticancer Agents

### 2.1. Parvovirus NS1

Oncolytic viruses replicate selectively in and lyse tumor tissues while showing non-productive infection of normal non-neoplastic cells [7]. Several species within the *Parvovirus* genus, in particular the rat parvovirus H-1 (H-1PV) and its mouse relative, the minute virus of mice (MVMp), have attracted high interest for their potential as anticancer agents. Parvovirus H-1 is an autonomous, single-stranded non-enveloped DNA virus of rat origin, capable of selectively killing a large panel of human cancer cells of different origins [8,9]. H-1PV and MVMp infection appear to be harmless in humans. The viral non-structural protein NS1, a 672-amino acid (aa) protein, is a key regulator of the parvoviral life-cycle. NS1 performs multiple roles like adenosine triphosphate binding and hydrolysis, site-specific DNA binding, DNA nicking, helicase, and promoter transregulation [10,11,12]. These properties enable NS1 to control a variety of processes that are necessary for progeny particle production including viral DNA amplification and gene expression [12]. The intracellular accumulation of NS1 protein owing to its bipartite nuclear localization sequence (NLS) between aa residues 194 and 216, is a major effector of the virus-induced cytotoxicity of the neoplastic cells. Furthermore, modification of specific residues (Thr-435 and Ser-473) of NS1 is important for cancer cell toxicity which is exerted, at least in part, by dysregulation of intracellular signaling pathways [13]. Cell death caused by NS1 was shown to be majorly induced by apoptosis and dependent on caspase-9-driven caspase-3 activation [14].

#### Mechanism of Action

**Figure 1:**
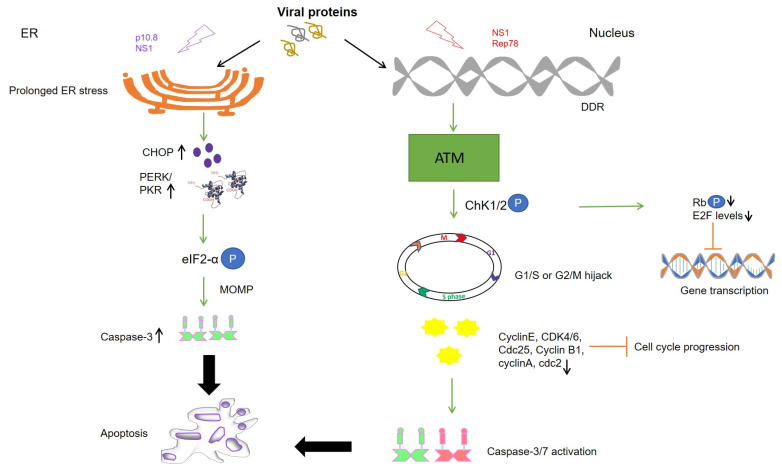
Schematic representation of different cell death pathways mediated by viral proteins. Levels of kinases are upregulated in cancer cells due to which phosphorylation and activation of viral protein residues (NS1 and p 10.8) lead to ER stress and DNA damage response (DDR) causing mitochondrial outer membrane permeabilization (MOMP), apoptosis, and cell death. Cell cycle arrest is mediated by activation of DDR (NS1) and downstream kinases like Ataxia telangiectasia mutated (ATM) and checkpoint kinases (Chk1/2). Cell cycle progression is inhibited at the G1/S or G2/M phase of the cell cycle as respective cyclins and CDKs are inactivated upon expression of the viral proteins mediated caspase activation (Rep78). At the same time, E2F inhibition is maintained by dephosphorylation of retinoblastoma protein (pRb) by Chk1/2, finally inhibiting the transcription of proto-oncogenes. Upward arrow↑—upregulation; downward arrow↓—downregulation; circled P in blue—phosphorylation.

NS1 is able to specifically target cancer cells since cellular factors mediating post-translational modifications are upregulated in transformed cells as compared to their normal counterparts. For instance, protein kinase C (PKC) isoforms causing phosphorylation of certain NS1 residues are elevated in cancer cells which results in stimulation of the cell-killing activity by the viral protein. NS1 from MVMp induces DNA damage response (DDR) by recruiting checkpoint kinase 2 (Chk2) for Ataxia telangiectasia mutated (ATM) phosphorylation which results in proteasomal degradation of cell division cycle (cdc) 25A, cyclin B1, p53 upregulation, and finally cell cycle arrest [10] (Figure 1). At the same time, p21 levels are maintained low by the viral protein during the early stages of infection to redirect the cellular machinery towards a more efficient replication. So far, reactive oxygen species (ROS) are considered to be a source of DNA damage by NS1 from H-1PV that contributes, at least partially, to both virus-induced DDR and cell cycle arrest [10]. The cytostatic potential of NS1 is mediated by an accumulation of cells in the G2 phase by upregulation of p21 and a block in cellular DNA replication [14]. The other mechanisms behind cytotoxic activities of the protein are still under investigation. Nevertheless, other studies have suggested the protein to form a complex with protein kinase II (CKII) which leads to the phosphorylation of components of the cytoskeleton. This, in turn, activates actin-binding protein, gelsolin, and suppresses signal-transduction by the Neural Wiskott–Aldrich syndrome protein (N-WASP) thereby causing cytoskeleton disruption [15]. H-1PV-induced cell death is facilitated by NS1-mediated p53 dependent or independent mechanisms through the accumulation of reactive oxygen species, mitochondrial outer membrane permeabilization (MOMP), DNA damage, cell cycle arrest, and finally, caspase activation [14]. H-1PV has already been recruited in phase II clinical trials for the treatment of pancreatic ductal adenocarcinoma and presently is in its evaluation stage [16].

### 2.2. Adeno-Associated Viruses (AAV) Rep78

AAVs, other members of the parvovirus family, are a group of non-enveloped, small, single-stranded DNA viruses, that rely on helper viruses like adenoviruses or herpesviruses for their efficient replication [17]. The autonomous and helper-dependent parvoviruses have unique biological properties in common. Members of both groups efficiently suppress tumor growth in animals through different proteins, irrespective of the mode of tumor induction [18]. The rep proteins, a family of multifunctional NS AAV proteins, are required for virus replication and gene regulation. AAV Rep78 was proposed to impair the utilization of cAMP pathway by helper viruses in HeLa cells and thereby inhibit productive replication of the helper virus [19]. In the early 1990s, AAVs were reported to inhibit carcinogen-induced simian virus 40 (SV40) DNA amplification [20] and carcinogen-induced resistance against methotrexate associated with amplification of the dihydrofolate reductase (DHFR) gene. Rep78 was found to interfere with both SV40 DNA amplification and herpesvirus replication [18]. Interestingly, Rep78 shares several properties with parvovirus NS1 like specific DNA binding, site-specific endonuclease, helicase, and ATPase activities, along with a cytostatic effect. The interaction of AAV Rep78 with p53 was suggested to be responsible for the observed protection of p53 in adenovirus-infected cells which is usually found to be degraded by the interaction of adenoviral E1B (early gene) with p53. By protecting p53 from ubiquitin-mediated degradation by adenovirus, the function of p53 as a cell cycle blocking agent is restored in the presence of Rep78 [21].

#### Mechanism of Action

It was earlier shown that Rep78-expressing cells display accumulation of the hypophosphorylated form of retinoblastoma protein (pRb) that leads to downregulation of E2 transcription factor (E2F) target genes, namely cyclin A, cdc2, and cyclin B [22]. Moreover, Rep78 inhibits the kinase activity of PRKX, a homolog of cAMP-dependent protein kinase A (PKA), and PKA itself, which results in the blockage of cAMP response element-binding protein (CREB)-dependent transcription in cervical cancer cells [19]. It was also found to be associated with the oncogenic transcription factor c-Jun and alter c-Jun-dependent transcription by inhibiting its binding to needed transcriptional partners/cofactors such as c-Fos likely through mechanisms of steric hindrance [23]. Furthermore, binding of Rep78 to the cell cycle regulatory phosphatase cdc25A prevents the latter access to substrates cyclin dependent kinase 1 (CDK1) and CDK2 thus resulting in the inactivation of CDKs that are required for continued DNA replication. However, the nicking activity of Rep78 together with the inactivation of Cdc25A is required to attain a strong, if not total, pRb inactivation [22]. It is possible that Rep78 induces DDR that causes pRb hypophosphorylation by Chk1/2 and forms a complex with E2F itself, eventually causing transcriptional inhibition of proto-oncogenes in cancer cells (Figure 1). Collectively, Rep78 has been observed to exert antiproliferative effects by blocking cell cycle in all of the phases and by inducing apoptosis independently of p53 via the caspase-3 dependent pathway [24]. In light of these findings, exploring the role of Rep78 in other cancer types and replicating the same in vivo may mark another milestone in virus-based anticancer therapy.

### 2.3. Human Herpesvirus Type 6 (HHV-6) Rep6/U94

HHV-6 is a double-stranded DNA lymphotropic β-herpesvirus existing as two closely related strains, namely HHV-6A and HHV-6B. The HHV-6/U94 gene, also known as Rep6, is highly conserved in both HHV-6A and B. It is a single-stranded DNA binding, exonuclease, helicase-ATPase protein which might be involved in DNA replication. It expresses at low levels during the early phases of viral replication [25]. U94 is known as a negative regulator of viral replication as it does not support productive viral replication in T-cell lines stably expressing U94. It accumulates in the treated cells and inhibits HHV-6A/B, HHV-7, and cytomegalovirus replication by blocking the virus cycle before genome replication [26]. U94 possesses a highly structural and functional similarity with AAV-2 Rep68/78 for which the viral protein has been studied for its involvement in cancer regulation [27]. Since U94 mRNA was detected in the peripheral blood mononuclear cells (PBMC) from latently infected healthy individuals, U94 was considered as a molecular marker of viral latency [28].

#### 2.3.1. Mechanism of Action

The viral protein was initially known for its ability to suppress Harvey (H)-ras-induced transformation in stably U94-expressing NIH 3T3 cell line [29]. Later, Ifon et al. [27] demonstrated the anticancer activity of U94 on human prostate cancer in vivo, as the development of human prostate cancer (PC3) cell line-derived tumor in nude mice was inhibited by treatment with a recombinant U94 protein. The anticancer activity of the U94 protein was possibly ascribed to Fibronectin 1 (FN1) upregulation and a concomitant Angiopoietin-like 4 (ANGPTL4) downregulation [27]. Indeed, increased levels of FN-1 are known to accelerate FN1-FN1 polymerization and FN1 binding to the PC3 cell surface which could be, at least in part, responsible for decreased clonogenicity in vitro and tumorigenesis in vivo. Moreover, the expression of SPUVE 23, a serine protease associated with increased malignant potential, was also observed to be downregulated in the recombinant U94-treated PC3 cell line [27]. Subsequently, U94 was also identified to impair tumor growth and invasion in glioma cells by promoting AKT/GSK3β signaling [30] and migration of oligodendrocyte progenitor cells (hOPC), thus highlighting its role in metastasis prevention [31,32]. Later, our group reported U94 ability to impair triple-negative human breast cancer cell (MDA-MB 231) migration, motility, invasion, and proliferation both in vitro and in vivo. The viral protein operates in MDA-MB 231 cells by downmodulating the activation of proto-oncogene Src and the downstream signaling pathways β-catenin/STAT3/cortactin/ARP2-3/Akt [33]. In a 3D fluid-dynamic environment, U94-positive cultures displayed β-catenin localization at the cell membrane, contrary to cytoplasmic localization of the same in transformed cells, which indicates a U94-triggered mesenchymal to epithelial transition (MET). At the same time vimentin, an epithelial to mesenchymal transition (EMT) marker highly expressed in MDA-MB-231 cells, was strongly down-modulated in cells treated with U94. Similarly, the expression of other EMT markers like TWIST, N-cadherin, Snail1, and matrix metalloprotease 2 (MMP2) was strongly inhibited, thereby supporting a role of U94 in mediating a MET of MDA-MB-231 cells. This was further confirmed in vivo as U94 inhibited tumor development of MDA-MB 231 xenografts in mouse models. Interestingly, similar to MDA-MB-231 cells, U94 also inhibited HeLa cells migration, proliferation, and colony formation both in vitro and in vivo through Src down-modulation [33]. U94 was found to reversibly arrest cell cycle in S-phase when transiently expressed in MDA-MB-231 cells [33]. Of late, our group demonstrated U94 to be a DDR inhibitor as it displays anticancer activity in MDA-MB-231 cells by downregulating DDR genes, cholesterol biosynthesis, and cell cycle, out of which cyclin-dependent kinase inhibitor 3 (CDKN3), Non-SMC Condensin II Complex Subunit G2 (NCAPG2), Ndc80 kinetochore complex component (NUF2), and High Mobility Group Box 1 (HMGB1), being the major ones. U94-mediated DDR inhibition likely occurs through downregulation of Bcl-2 and upregulation of Bax-, Bad-levels, Poly (ADP-ribose) polymerase (PARP) cleavage, and caspase-9-exerted apoptotic cell death via intrinsic apoptotic pathway activation [34]. The anticancer function of the viral protein was also investigated and confirmed in other triple-negative human breast cancer cell lines like MDA-MB 468 and BT-549 cells. In particular, U94 worked in synergy with DNA damaging drugs such as cisplatin and doxorubicin to attack tumor cells [34].

#### 2.3.2. Role of U94 in Blocking Angiogenesis

**Figure 2:**
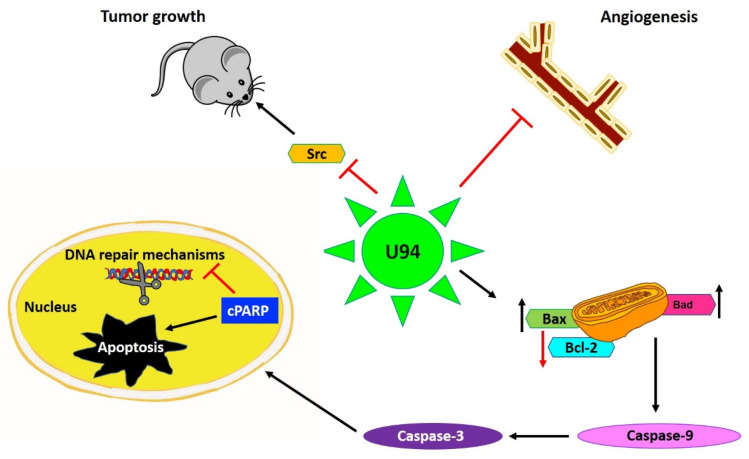
U94 regulates different mechanisms to exert its activities mainly by downregulation of proto-oncogene Src, resulting in inhibition of cancer cell proliferation both in vitro and in vivo. Secondly, U94 was found to suppress angiogenesis ex vivo by suppressing the vessel formation. The viral protein upregulates pro-apoptotic genes like Bax and Bad levels and downregulates anti-apoptotic Bcl-2 levels culminating in Caspase-9 activation. Finally, the cleavage of Poly (ADP-ribose) polymerase (PARP) results in inhibition of DNA repair and mediation of intrinsic apoptosis in cancer cells. Upward arrow↑—upregulation; downward arrow↓—downregulation.

Previous work by our group showed that HHV-6 infection of endothelial cells (EC) resulted in a strong inhibition of angiogenesis in vitro and ex vivo. In the latter condition, treatment of rat aortic rings with U94 rendered them insensitive to vascular endothelial growth factor (VEGF)-induced vasculogenic activity [26]. Later, we identified that U94-expressing tumor xenografts of mouse origin displays impaired vasculogenesis [33]. Surprisingly, human umbilical vein endothelial cells (HUVEC) co-cultured with U94-expressing MDA-MB-231 cells lost their ability to perform angiogenesis or migrate in vitro. Consequently, the secretome of U94-expressing MDA-MB 231 cells was used to test its activity on HUVECs angiogenic activity. As expected, the secretome derived from U94-expressing breast cancer cells was found to completely inhibit angiogenesis of HUVECs in vitro [33]. This finding was strongly suggesting on the involvement of a U94-induced soluble factor in inhibiting EC angiogenesis. Recently, HHV-6A has been found to induce the expression of the non-classical class I Human leukocyte antigen-G (HLA-G) molecule in primary human mesothelial cells as a mechanism for viral immune-escape [35]. It is worth noting that HHV-6 was found to promote HLA-G expression by activating the activating transcription factor 3 (ATF3), a member of the basic leucine zipper domain (bZIP)/CREB proteins, which can interact directly with the HLA-G promoter thereby stimulating the HLA-G production [36]. For this reason, the involvement of HLA-G in sustaining the anti-angiogenic activity of U94 on ECs was investigated and recently confirmed [36]. Altogether, these results highlight the capability of U94 to mediate the impairment of cancer progression through a “two compartments” activity, thus providing anticancer therapeutic benefits not only in terms of antiproliferative and lytic effects on different tumor cells but also inhibiting the neovascularization process needed for cancer cell growth and metastatization (Figure 2).

## 3. Why Avian Viral Proteins as Anticancer Therapeutics?

Avian viruses are known to cause morbidity and mortality including diseases like viral arthritis, hepatitis, respiratory syndromes, immune suppression in species like goose, ducks, turkeys, pigeons, raptors, and quails, but not in humans. The economy and poultry industry have been affected in several countries due to these viruses. However, viruses derived from such sources that do not circulate extensively in the human population represent a potential source of viral proteins able to bypass any pre-existing immunity [37]. Due to which they might prove to be safer and effective candidates to be utilized in cancer therapy with lesser side effects as compared to conventional viral vectors with efficacy and processing risks. Interestingly, most of the avian viral proteins possess nuclear localization property that makes them effective in targeting cellular mechanisms. In the next section, we have summarized some of the newly emerged avian viral proteins which have recently been investigated for their potential functions and may prove to be promising anti-carcinogenic agents in the future.

### 3.1. Chicken Anemia Virus (CAV) Apoptin

CAV, a member of the genus *Gyrovirus*, is an etiological agent of chicken infectious anemia known to cause immunosuppression in young chickens and compromise immune response in older birds. CAV mainly infects hematopoietic cells including bone marrow-derived cells [38,39], myeloid progenitor cells, and T-lymphocyte precursor cells [40]. VP3, a 121 amino acid-long structural protein from CAV is known for its property to induce apoptosis and viral cytotoxicity in host cells, hence the name apoptin [41]. The C-terminal domain of apoptin contains a bipartite nuclear localization sequence (NLS, aa 82–88 and 111–121) and a secondary nuclear export sequence (NES, aa 97–105) and together these motifs confer to the protein a nucleocytoplasmic shuttling activity [42,43]. Apoptin is known for its multimerization and nucleus retention activity in human transformed or tumor cells from tissues of endodermal, ectodermal, and mesodermal origin. It interacts with cellular proteins like anaphase promoting complex (APC/C) and transports the latter from cytoplasm to nucleus to be deposited in promyelocytic leukemia (PML)-nuclear bodies, whereas the same remains cytoplasmic in normal human cells [42]. Apoptin is commonly phosphorylated at Thr-108 in osteosarcoma (U2OS, Saos-2), lung carcinoma (H1299), colon carcinoma (HT29), cervical carcinoma (HeLa), hepatocellular carcinoma, and other transformed cells [42,44,45,46]. Although phosphorylation status is not necessary for nuclear localization, it does play a role in determining the toxicity of the viral protein. Instead, mutations in leucine-rich sequence (LRS, aa 33–46) cause reduced nuclear accumulation of apoptin in cancer cells [42]. In normal cells, apoptin has been shown to be localized in the cytoplasm, aggregated, and eventually degraded [45]. Therefore, apoptin can selectively kill various human tumor or transformed cells with little cytotoxic effect in normal cells.

#### 3.1.1. Mechanism of Action

**Figure 3:**
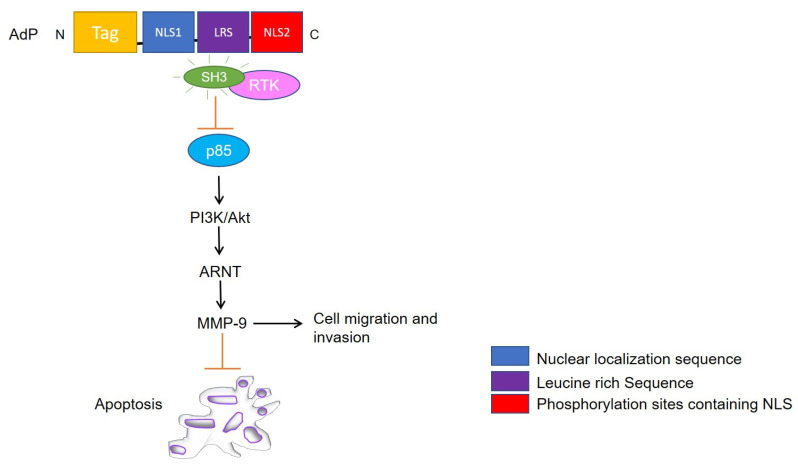
Schematic representation of apoptin derived peptide (AdP) targeted signaling pathways. AdP binds to SRC- homology 3 (SH3) domain of tyrosine kinases and deactivates PI3K/Akt pathway followed by Aryl hydrocarbon nuclear translocator (ARNT) signaling and MMP-9 suppression leading to inhibition of cell migration along mediating apoptosis.

Apoptin triggers caspase-dependent cell death via the intrinsic apoptotic pathway [47,48,49] independently of p53, but requires pro-apoptotic transcriptionally active p73 isoforms from p53 family [50,51]. Like parvovirus NS1, DDR signaling plays a key role in nuclear localization and apoptosis induction by apoptin [52]. In a study by Kucharski et al. [53], the authors show that the inhibition of kinases Chk1 and Chk2 in non-small cell lung adenocarcinoma (NSCLC) results in cytoplasmic re-localization of apoptin. Therefore, the phosphorylation of residues T56 and T61 is relevant in regulating the localization and apoptotic activity of the protein. Other studies have found apoptin to trigger the nuclear accumulation of the related kinase Akt in prostate cancer [54] and PKCβ1 in colon cancer cells via interaction with the N-terminal region of the viral protein [55]. Recently, PKCβ1 was shown to phosphorylate apoptin in multiple myeloma cell lines [55], thereby indicating PKCβ1 to be a tumor-specific target responsible for sensitizing cells to apoptin [56,57,58]. Apoptin also drives translocation of the transcription factor nuclear hormone receptor (Nur) 77 from the nucleus into the cytoplasm, where it causes mitochondrial outer membrane permeabilization and induces cytochrome C (cyt c) release mediating intrinsic cell death pathway [47,48] (Figure 3). Other studies have shown that apoptin interacts with and inhibits Abl/BCR-Abl1 kinase and downstream targets, like STAT5, CrkL, and c-myc in chronic myeloid leukemia (CML) [59]. Jangamreddy et al. [60] designed an apoptin-derived decapeptide (AdP, aa 81–90) as an alternative therapeutic agent, which acts as a negative downregulator of BCR-Abl1 and is capable of downstream targeting c-myc with comparable efficacy to full-length apoptin.

#### 3.1.2. AdP

AdP is a 5.2 kDa hydrophilic, highly soluble peptide, consisting of four parts: a penetrating peptide Tat (for entering facilitation), the core NLS1, the NLS2 sequence, and a flexible connection (LRS) between NLS1 and NLS2 [61]. The peptide was tailored to facilitate targeting to cancer cells and nuclear accumulation. Owing to its small size, the peptide was designed to exhibit reduced immunogenicity and strong antitumor activity against glioma cells. The molecule led to reduced heat shock protein 70 (HSP70) mRNA and protein levels in tumor cells and proved to be more effective in mediating glioma cell apoptosis than apoptin itself both in vitro and in vivo [61]. Song et al. [62], postulated the potential mechanisms for glioma inhibition to be linked with the interaction of AdP with heat shock element-SRC homology 3 (HSE-SH3) domain, inactivation of RTK/PI3K/Akt pathway, and MMP-9 suppression (Figure 3). However, compared to apoptin, AdP increases apoptosis in human astrocytes, but to a lower extent than in glioma cells [61]. Interestingly, down the line, Hou et al. [63] demonstrated that recombinant apoptin (GST tagged apoptin, chemically modified by folic acid for entering into the cells) inhibits the growth of breast cancer cells likely by triggering apoptosis. They demonstrated that recombinant apoptin inhibits proliferation and induces apoptosis in vitro following similar molecular mechanisms as apoptin by facilitating the expression levels of Bax, Cyt c, p-Akt, and p-Nur77. Another study by Zhou et al. [64], showed that AdP inhibited cell viability in cisplatin-resistant gastric cancer cells, without affecting normal cells by PI3K/Akt/ARNT signaling pathway. Overall, AdP causes an increase in the G2/M phase population leading to apoptosis and priming the cells sensitive to chemotherapy likely due to decreased expression of AKT, p85, and their phosphorylated forms in both therapy sensitive and resistant cells. Collectively, these findings suggest that the apoptin derived peptide could be used in combination with other drugs and targeted for different kinds of cancer therapy, provided the safety of normal cells has been assured.

### 3.2. Avian Reovirus (ARV) p17

ARV belongs to the genus *Orthoreovirus* in the *Reoviridae* family. It has a double-stranded 10-segmented RNA genome encoding for at least eight structural and four NS proteins. ARV p17 (p17) is a 17-kDa non-structural protein encoded by the S1 gene and contains 146 aa [65]. The S1 genome segment of ARV contains three open reading frames that translate into p10, p17, and σC proteins, respectively. P10 displays membrane destabilization activity [65,66,67], whereas σC is known to be a cell attachment protein [68] capable of inducing apoptosis [69,70,71] and p17 as a nucleocytoplasmic shuttling protein with an unknown function. Previous studies have shown that p17 has a basic region, spanning from aa 119 to 128 (IAAKRGRQLD), which is similar to the functional monopartite NLS of the c-Myc protein and is highly conserved in different ARV isolates [72]. A monopartite-type functional NLS near the C terminus of p17 is necessary for nuclear import. NLS interacts with the nucleoporin translocated promoter region (Tpr) localized within the nuclear pore complex and causes suppression of Tpr thereby activating cell cycle regulators like p53, Phosphatase and tensin homolog (PTEN), and p21. The activation of CDK inhibitors causes downregulation of both PI3K/Akt/mTOR and ERK signaling pathways [73,74]. Previous studies have shown that p17 causes retardation of cell growth by deactivation of mTORC1 and downstream protein synthesis through activation of the p53 pathway [75]. In a recent report, Chiu et al. [74] showed that p17 contains a NES spanning from aa 19 to 26 (LSLRELAI), which is required for interaction with the heterogeneous nuclear ribonucleoprotein (hnRNP) A1 and serves as a carrier in mediating nucleocytoplasmic shuttling of the viral protein. While lamin A/C mediates p17 nuclear import, p17-hnRNP A1 carrier-cargo complex causes downregulation of Tpr by direct interaction with lamin A/C and Tpr.

Altogether, p17 has been observed to induce cell growth retardation, cell cycle arrest, and host cellular translation shutoff by suppression of CDK1- and polo-like kinase (PLK1)- like signaling pathways and regulation of the p53/PTEN/mTORC1-like pathway [76]. Since p17 is also known to induce autophagy and trigger protein kinase RNA (PKR)-activated signaling, it activates the innate immune system and can mount the immune response against tumors. In summary, p17 appears to divert the cellular machinery required for normal cell-cycling processes, including ATM/Chk1/2 signaling pathway [77] to allow virus replication via induction of cell cycle arrest and cellular translation shutoff [75,76,78]. Secondly, p17 positively regulates PTEN, AMP-activated protein kinase (AMPK), and PKR/eIF2 signaling pathways accompanied by downregulation of Akt and mTORC1, thereby triggering autophagy [78]. Autophagy-induced activity of p17 has been observed to be mediated by p17 nuclear localization, as the former is delayed and viral replication is affected when the protein could not enter into the nucleus [79].

#### 3.2.1. Role in Cell Cycle

P17 exhibits cell growth inhibition and cell cycle retardation in multiple cell lines like African green monkey kidney epithelial cells (VERO), chicken fibroblasts (DF-1), human adenocarcinoma (SW620), HeLa, and human lung cancer (A549), along displaying reduced tumor size in vivo [76]. P17 acts on inhibiting CDK1 in two different manners: first, by suppressing the phosphorylation of CDK1 via suppression of kinases like PLK1 and CDC25c; and secondly, by competing with cyclin B1 to bind CDK1 leading to CDK1 retention in the cytoplasm. Prevention of the cyclin B1/CDK1 complex formation in the nucleus leads to G2/M cell cycle retardation [76]. Moreover, p17 expression is also responsible for p53 and PTEN phosphorylation by impairing the targeting ability of the corresponding E3 ubiquitin ligase, namely mouse double minute 2 homolog (MDM2) [80]. Enhancement of p53 interaction with cyclin H mediates suppression of cyclin-associated kinase (CAK) activity by p17 and dissociates the CDK7/Cyclin H complex. The same study reported a particular motif in the p17 protein spanning from aa 140 to 143 (WXFD) and conserved residues at positions D113 and K122, as critical for CDK2 and CDK6 binding [74]. The p17 binding to cyclins via conserved motifs is a peculiar property of most of the renowned tumor suppressor proteins. To conclude, p17 suppresses the formation of CDK1/cyclin A2, CDK2/cyclin E1, and CDK6/cyclin D1 complexes by directly binding to CDK, cyclin, or CDK/cyclin complexes, benefitting viral replication [74]. The two prime pathways behind this activity are PI3K/AKT- and Tpr/p53/PTEN/ERK-dependent inhibition of the mTORC1 pathway.

#### 3.2.2. Role in Angiogenesis

**Figure 4:**
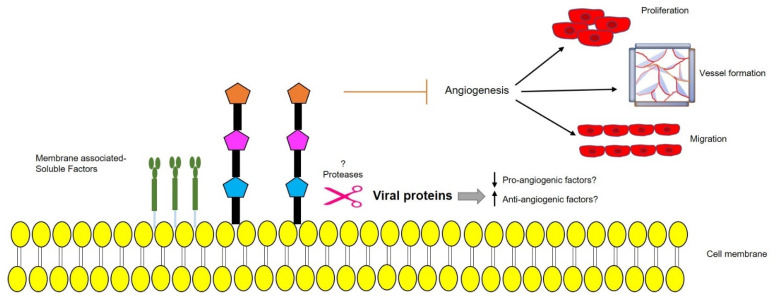
Schematic representation of a possible protease-mediated mechanism, triggered by ARV p17, leading to secretion of membrane-bound anti-angiogenic factors that prevent endothelial cells (EC) migration, proliferation, vessel formation, and angiogenesis. Upward arrow↑—upregulation; downward arrow↓—downregulation.

We recently showed that p17 is also able to inhibit motility, migration, and angiogenesis in human macrovascular (HUVEC) and microvascular ECs (HMVEC). Treatment of ECs with recombinant GST-p17, or over-expression of p17 at the intracellular level by nucleofection of a p17-expressing plasmid, led to the suppression of tube-like formation on Matrigel, cell migration, and sprouts generation in a 3D spheroid assay. Interestingly, p17 was found to downregulate EC angiogenic activity in the presence of different mitogenic stimuli like VEGF-A and basic fibroblast growth factor (FGF-2), thereby confirming its wide anti-angiogenic spectrum of activity. The anti-angiogenic activity mediated by p17 was also demonstrated ex vivo and in vivo by aortic ring assay and chick chorioallantoic membrane (CAM) assay, respectively, where the viral protein proved to suppress the number of neovessels formation while remaining non-toxic to the normal tissues [81]. Furthermore, we observed the secretion of a well-known tumor suppressor molecule, namely dipeptidyl protease 4 (DPP4), in the supernatants of p17 expressing HUVECs and HMVECs (Figure 4). Further studies may confirm the pathways responsible for upregulation of soluble-DPP4 in the presence of p17. Whether pro-angiogenic factors are downregulated due to the secretion of anti-angiogenic factors by ARV p17 or vice versa, remains to be solved. Up-regulation of protease levels in the presence of p17 responsible for the secretion of membrane-associated factors as DPP4 is another field yet to be explored.

### 3.3. Muscovy Duck Reovirus (MDRV) p10.8

Other viral proteins from avian species and homologous to ARV p17 serve important cell growth regulatory functions and might exert anticancer activities in tumor cells. MDRV is another member of the genus *Orthoreovirus*, an important poultry pathogen that is involved in several diseases including viral arthritis, pericarditis, hepatitis, respiratory syndromes, and sudden death. Ducklings infected with this reovirus were first reported in 1950 and MDRV was first isolated in 1972 [82]. Its genome consists of 10 double-stranded RNA segments which can be separated into three size classes: L (large), M (medium), and S (small). Like other avian reoviruses, MDRV appears to evolve mechanisms that alter the physiology of host cells during infection to increase its replication. MDRV was first appeared to induce autophagy in chicken fibroblasts via suppression of mTOR phosphorylation and marked increased levels of Microtubule-associated protein 1A/1B-light chain 3 (LC3-II) induced by a NS protein named σNS [83]. Another MDRV protein, named p10.8, is coded by the S4 gene sequence and is found highly conserved suggesting that p10.8 plays an important role in virus-host interaction [82]. This polypeptide has no significant sequence similarity to other known proteins, so its amino acid sequence offers no clues about its function [84]. Similar to ARV p17, p10.8 can localize to the nucleus independent of the host cell type based on a signal mediated import. MDRV p10.8 has an aromatic amino acid-rich NLS which enables it to pass through the nuclear pore complex and a leucine-rich NES [84]. Recently, MDRV p10.8 has garnered attention due to its apoptosis-inducing ability in DF-1 and VERO cells [84].

#### Cell Cycle Arrest

Like ARV σC, p10.8 induced apoptosis is associated with ER stress through unfolded protein response-mediated BIP/IRE1/XBP1 pathway [85,86,87]. The viral protein is known to dissociate the BIP/IRE1 complex and increase the phosphorylated form of inositol-requiring enzyme 1 (IRE1) to activate X-box binding protein 1 (XBP1) as indicated by the increased mRNA levels of binding immunoglobulin protein (BIP), XBP1, C/EBP homologous protein (CHOP), and caspase-3 [87]. Another study revealed that p10.8 induced cell cycle arrest at the G0/G1 phase in DF-1 cells by dissociating BIP from protein kinase R-like ER kinase (PERK) and increasing phosphorylated PERK and eukaryotic initiation factor 2 subunit 1 (eIF2α) levels [82]. Taken together, the viral protein increases the protein expression of BIP, p-PERK, p-eIF2α, CHOP, cleaved-Caspase 12, and cleaved-Caspase 3, thus indicating that the p10.8 protein induces ER stress-mediated apoptosis (Figure 1). This finding implies a cell cycle-regulated role of p10.8 in inducing apoptosis-related cell death. Furthermore, high levels of kinases in tumor cells may promote high specificity of action for p10.8. Recently, it has been demonstrated that both MDRV p10.8 and ARV σC can mediate CDK4 ubiquitination by stabilizing Cdc20 with the aid of molecular chaperones, chaperonin containing TCP1 subunit 2 (CCT2), and 5 (CCT5) [88]. On the other hand, the fact that both p10.8 of MDRV and σ1s of mammalian reovirus can localize to the nucleus and cause apoptosis of infected cells suggests that σ1s and p10.8 may be functionally related [89]. Nuclear import of p10.8 mediates activation of p53 in VERO cells possibly via suppression of nucleoporin Tpr as with ARV p17. This, in turn, leads to the activation of extrinsic cell death via activation of the Fas/caspase 8/caspase 3 pathway [85]. This property of the protein to modulate different apoptotic and cell cycle control pathways may make it a suitable candidate for targeting tumor cells mostly because of its similarity with ARV p17 in PKR activation and nuclear localization. However, based on these primary studies, further characterization of the protein is required in cancer cells along with addressing its cytotoxic activity on normal cells.

### 3.4. Newcastle Disease Virus (NDV) F Protein

Newcastle disease virus (NDV) is an avian paramyxovirus, a member of the Avulavirus genus with a negative single-strand RNA genome. It is one of the most well-researched oncolytic viruses for its activity against all kinds of cancer cell lines from ecto-, endo-, and mesodermal origin, but not normal cells [90]. It has displayed an impressive safety profile in phase I and II human clinical trials. NDV binds to the sialic acid (Sia) receptor on host cells and, therefore, can infect a broad range of cell types. Different receptor isoform expression patterns between cell types contribute to the selection of cancer cells like HeLa by NDV over normal cells like BHK fibroblasts [91,92]. NDV can achieve oncolysis via activation of the extrinsic or intrinsic apoptosis, activation of PERK kinase followed by caspase-12, and secretion of cytokines like tumor necrosis factor–α (TNF-α) amongst various others, from the infected tumor cells [93]. Engineered NDV vectors expressing apoptin [94], immune checkpoint blockades like anti-CTLA-4, anti-PD-1, anti-PDL, and cytokines like IL-2 [95] and influenza virus NS1 [96] trigger oncolytic cell death in tumor cell lines from lung and liver, tumor-bearing mice [94], and apoptosis-resistant cells, respectively [96].

The F protein from NDV is a class I viral membrane fusion protein present as a trimer in the virion where the cleavage site of the F protein is known to be responsible for virulence and the formation of syncytia [92]. Both Fusion (F) and Hemaglutinin-Neuraminidase (HN) proteins expressed on the surface have been studied to interact and fuse with host cellular membranes. Upon adsorption of HN to its cellular receptors, F protein undergoes a conformational change which triggers the release of fusion peptides to fuse the viral and cellular membranes [97]. Intracellular insertion of viral HN and F surface antigens were reported to induce a strong inflammatory response with the secretion of cytokines, chemokines, and type I interferons (IFN). This, in turn, modulates tumor cell surface markers and induces downstream apoptosis [93]. However, Liu et al. [92] lately reported that that the F protein plays a major role in NDV-induced oncolytic effect on xenograftic mice from H22 and 4T1 cell lines, possibly via mtorc1 inhibition but it remains to be confirmed in further studies. Like ARV p17, NDV F protein is also postulated to induce autophagy by upregulating autophagy related 5 (ATG5), beclin-1, and microtubule-associated proteins 1A/1B light chain 3B (MAP1LC3B) like markers.

## 4. Old World (OW) Alphaviruses

Alphaviruses belong to the family of *Togaviridae*. They are non-segmented, positive-stranded RNA, enveloped viruses with an icosahedral structure [98]. OW alphaviruses include sindbis virus (SINV), semliki forest virus (SFV), and chikungunya virus (CHIKV), which share many common characteristics. Nearly all the alphaviruses are arthropod-borne and are transmitted to their vertebrate hosts by mosquitoes [99]. The naturally occurring OW alphaviruses are relatively milder in humans and severely infect species like cattle, birds, and horses. The oncolytic SINV, SFV, and other alphavirus vectors have been reviewed in [100]. Here, we focus on SINV as a representative member of the alphavirus family and involvement of its structural proteins in mediating cytotoxicity in cancer cells.

### 4.1. Mechanism of Action

SINV has a genomic RNA of 11.7 kb and is primarily known to target lymph nodes. It is transmitted to birds and mammals by mosquito bites [101] and subsequently spreads throughout the body via the bloodstream [102]. In humans, SINV infection is considered to induce no symptoms or only mild symptoms (fever, rash, and arthralgia) [98] suggesting low infectivity and viral replication in normal tissues. SINV was shown to induce cytopathic effects in ovarian and cervical cancer cells without affecting normal keratinocytes. It was found to be stable in the bloodstream and effective in targeting remote tumors as observed by regression of cervical tumor xenografts in SINV infected mouse models [103]. The virus induces cell death in human squamous carcinoma (HSC-3) cells through apoptosis related to caspase-3, 9, cytochrome c, Nuclear factor kappa B (NF-kB), NF-kB inhibitor (IkBa), and IkB kinase (Ikk) modulation [104]. The extensive virus-induced activity was also testified by neuroblastoma regression in nude mice upon intratumoral and intravenous administration of SINV AR339 strain [105]. Additionally, SINV infection regulates the cell cycle progression in HeLa cells by accumulating cells in the S phase and relatively shortening the G1 phase by exerting upregulation of cyclin E, Cdc25A, and CDK4/6 levels. The virus causes downregulation of p21 during the early phases of infection and facilitates quick entry into the S phase. However, the S phase was found to be arrested during the later stages of infection when SINV starts to downregulate cyclin A levels [102].

### 4.2. SINV E1 and E2

**Figure 5:**
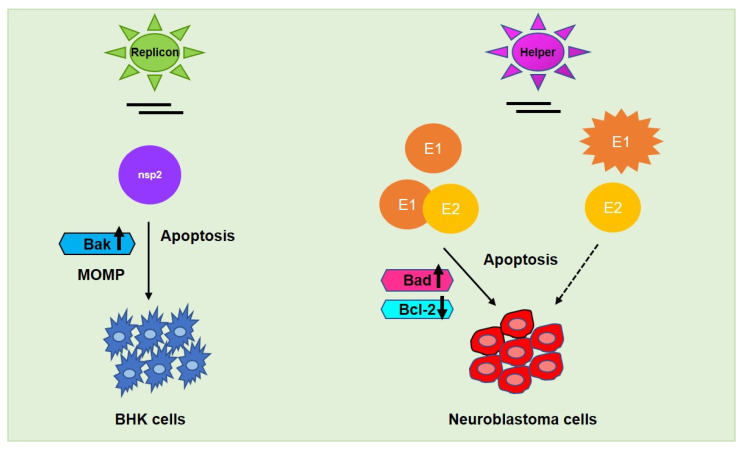
Alphaviral induced apoptosis facilitated by its respective non-structural protein nsp2 (encoded by replicon vector) in baby hamster kidney (BHK) fibroblasts and structural proteins E1/E2 (encoded by helper vector) in neuroblastoma and other cancer cells. E2 alone—could not mediate the same effect as E1 alone—or E1-E2 heterodimer in metastasis regulation whereas UV-inactivated SINV may commence oncolysis in the presence of E1, specifically in neuroblastoma cells. Upward arrow↑—upregulation; downward arrow↓—downregulation.

The alphavirus genome encodes six structural proteins (capsid, 6K, and three surface glycoproteins, E1, E2, and E3) and four non-structural proteins, nsP1-4, which are components of the viral replicase and transcriptase [106,107,108]. nsP2 is known for its cytotoxicity since it is able to shut down cellular transcription, translation, and induce apoptosis in BHK cells [109]. However, there has not been shown any strict correlation between nuclear localization of nsP2 and cytotoxicity since the nsP2-mutant replicons retained nuclear localization while remaining non-cytotoxic. The SINV envelope protein E2 is responsible for cellular-receptor binding and E1 is required to promote the fusion between viral particles and cell membrane [107]. It was earlier shown that alphaviral structural proteins contribute to cell death by apoptosis as virus replicon particles (VRP) lacking E1 and E2, showed a delay in caspase activation thereby concluding that structural proteins contribute to apoptotic activity in cancer cells [109]. Both of the viral structural proteins, in addition to nsP2, have been shown to play a role in alphavirus-induced apoptosis [110]. SINV induced apoptosis is caspase-8 dependent and mediated by Bad [108]. The expression of SINV structural envelope proteins, either E1 or E2, led to apoptosis in rat prostatic adenocarcinoma (AT-3) cells [109]. However, a single amino acid change in the SINV E2 from Q55 to H55 conferred both neurovirulence in mouse neuroblastoma (N18) cells and the ability to kill AT-3 cells expressing bcl-2, likely due to the alteration of the interaction between E2 and bcl-2 [111]. Later, Hurtado et al. [112] investigated the functional amino acids of the E2 envelope protein in SINV and identified that the change of amino acid E70 to K70 suppressed the metastasis-targeting ability of the protein, possibly due to inhibited interaction between E2 and E1 in the vector spike confirmation. In a recent study, Saito et al. [98] demonstrated that E1 induced higher cytotoxicity than E2 in human neuroblastoma cell lines (NB69, NGP, and RT-BM-1). Moreover, E1 and E2 heterodimers or E1—but not E2 alone—was able to exhibit cytotoxicity in neuroblastoma cells (Figure 5). Furthermore, in the presence of E1, the UV-inactivated SINV induced cytotoxicity specifically in human neuroblastoma cells but not in normal human fibroblasts, affirming E1 as a potent therapeutic agent.

## 5. Administration Tools

Conventional delivery methods for transient gene expression like transfection, lipofection, nucleofection, or electroporation along with viral delivery methods like lentiviruses, adenoviruses, baculoviruses are commonly used for intracellular administration of almost all the listed viral proteins, particularly apoptin. For instance, integration-deficient lentiviral vectors (IDLV) were implicated for delivery of Rep78 in HEK 293 cells [113] and flippase (Flp)-derived recombination system to attain the stable expression of NS1 in HeLa cells [14]. Viral vectors co-expressing enhanced green fluorescence protein (EGFP) like Retroviral-vector expressing Rep78 [22] and HSV-1 amplicon vector expressing U94 [33] were used to ameliorate the need of a drug resistance gene and aid cellular tracking. Furthermore, Sindbis viral vectors have been produced by co-electroporation of in vitro-transcribed RNAs from replicon (replicase and viral subgenomic promoter sequences) and helper plasmids (viral subgenomic promoter, capsid, and envelope protein sequences) to administer nsp2 and E1/E2 proteins, respectively (Figure 5) to minimize collateral effects in vitro whereas intraperitoneal injection of SCID mice with Sindbis vectors (~10^6^ TU) resulted to be a successful therapy in vivo [112]. Similarly, NDV chimeric rClone30 vectors were designed by recombination of Clone30 lentogenic strain with F and HN genes from Anhinga mesogenic strain to achieve desired oncolytic activity in the absence of hazardous consequences associated with virulent velogenic strains [92].

However, to overcome targeting and internalization limitations associated with the use of viral or non-viral vectors, cell-penetrating peptides (CPP) proved as an effective approach towards accelerating the functional activity of anticancer molecules. Small CPPs, such as Tat, are used as ideal tools for the delivery of apoptin and AdP in vitro and in vivo. Other cationic fusion systems, namely human-derived CPP 10 (hpp10), protein transduction domain 4 (PTD4), VP1 from chicken anemia virus (CVP1) were also found to penetrate tumor tissues in vivo [13,61]. MT23, a CPP screened by phage display in B16 melanoma cells, was shown to not able to enter normal human cells but only melanoma cells, and MT23- fused apoptin significantly inhibited tumor growth and induced cell apoptosis in B16 tumor-bearing mice. [114]. Azurin-p28, a tumor-homing anionic peptide known for its antitumor activity was fused with apoptin showed selective cytotoxicity towards breast cancer cells without hampering normal cells. p28 is listed in two clinical phase I trials for the treatment of solid p53 tumors [115].

Purified recombinant proteins are another efficient means for targeted delivery to the cells without cytotoxic effects. Different fusion tags are conjugated to the peptides or proteins to aid the purification and transportation into the cells. Recombinant GST conjugated Apoptin modified by folic acid facilitated its delivery to human breast cancer cells [63]. Moreover, Rep78 as a fusion protein with Maltose binding protein- (MBP-) was successfully administered in HeLa and SW13 cells, as well as C127 murine fibroblasts [116]. Similarly, recombinant U94 as a fusion protein with a His-Tag [26] and ARV p17 with either Thioredoxin A conjugated His- (TrxA-His-) [73] or GST-tag [81] facilitated its cellular entry to regulate multiple processes. GST-ARVp17 also traversed the allantoic membrane in chick embryos assay. The His- and Flag-tagged synthetic peptides containing NLS or NES sequences of ARV p17 were shown to interact with Tpr and hnRNP A1, respectively [74].

Transduction using the peptides fused to proteins has an advantage in that entry is rapid and concentration dependent, and works with several cell types. However, unraveling new approaches to deliver the viral proteins efficiently to human systems will remain a constant hustle. A safer and robust protein delivery using gag-driven virus-like particles (VLP) can deliver proteins both to the surface and the interior of cells. Kaczmarczyk et al. [117] presented a safer alternative to physical and chemical delivery methods in which VLPs derived from an unrelated avian influenza retrovirus are used to effectively deliver protein to the cells. Moreover, apoptosis-inducing ligands can be displayed on the surface of VLPs to generate appropriate response inside the cells.

Finally, to achieve long-term efficacy, nanocarrier-based delivery systems have been utilized as therapeutic cargoes. The use of microparticles like lipid nanoparticles to drive viral mRNA in the development of the SARS CoV-2 vaccine has opened new frontiers for the administration of viral components to the human system [118]. Moreover, extracellular vesicles that could be delivered intranasally or intravenously meeting stability, compatibility, and potency needs, can be further improved as drivers of protein therapy to acquire desired regulatory effects.

## 6. Conclusions and Future Directions

Viruses that normally live in and among us serve as genetic blueprints that enable them to make biologically active molecules. Proteins from human and non-human viral sources may prove useful as therapeutic molecules to treat cancer by stimulating extrinsic (caspase-mediated) or intrinsic (mitochondrial) apoptosis pathways and targeting cell cycle regulation (Table 1). Since safety issues are one of the concerns in bringing engineered viruses to clinical applications, unravelling viral components that serve cancer-suppressing roles sounds a promising alternative. Viral proteins may act as powerful tools for controlling cell biology and provide the basis for developing new therapeutic drugs against cancer. Cancer cells may develop resistance to drugs and neutralizing antibodies to oncolytic vectors (OV) but viral proteins provide a long-term cure by inducing deeper and faster molecular and cytogenetic responses, and dosage-dependent effects. Viral proteins with novel functions will greatly improve the mechanistic knowledge about their activity and help us to design small, simple, and non-immunogenic polypeptides that are still able to achieve the desired biological activity. Further studies may shed light on the use of viral proteins in combination with other proteins or anticancer agents since they are able to regulate multiple pathways involved in cell growth perturbation. The viruses reported in this review, irrespective of their source and origin, focus mainly on the proteins derived from the former known for their action against cancer to eliminate the need for a whole virus. More viruses and respective proteins are currently under investigation for finding biologically active epitopes to add to their safety and therapeutic potential.

Viral proteins like HHV-6 U94 and ARV p17, other than their participation in cancer suppression are also responsible for regulating vessel formation by modulating the levels of soluble factors that cause suppression of angiogenesis. These findings pave the way for a successful cancer therapy where minimalist viral proteins or peptides will represent future drugs for humans.

## Figures and Tables

**Table 1:** Binding partners and their role in cancer growth suppression by viral proteins.

Protein	Host	Function	Mode of Action	Binding Partners	References
NS1	Rat	Endonuclease, Helicase, ATPase	DDR, cell cycle arrest, intrinsic apoptosis	PKC, CKII	[10,15]
Rep78	Human	Endonuclease, Helicase, ATPase	Cell cycle arrest, apoptosis	PKA, p53, c-jun, cdc25A	[19,21,22,23]
U94	Human	Exonuclease, Helicase, ATPase	Intrinsic apoptosis, sHLA-G release, src downregulation	TATA-binding protein	[25]
Apoptin	Chicken	Apoptosis	Intrinsic apoptosis	PML, APC/C, PKC, Akt, Nur77, BCR-Abl1	[42,47,55,63]
AdP		Apoptosis	MMP-9 inhibition	HSE-SH3	[62,64]
P17	Chicken	Autophagy? (?—unconfirmed)	Autophagy, cell cycle arrest, sDPP4 release	hnRNPA1, lamin A/C, Tpr, CDK1, cyclin A/D/E	[74,77]
P10.8	Duck	Apoptosis	Extrinsic apoptosis, cell cycle arrest	CCT2/5	[88]
F	Birds	Fusion, Virulence	Autophagy? (?—unconfirmed)	α-2,6 Sialic acid receptor	[92]
nsP2	Birds, Horses, Cattle	Nucleoside triphosphatase, helicase	Apoptosis, ER stress response, cell cycle arrest	RBP1 (RNA polymerase II)	[99]
E1/E2	Birds, Horses, Cattle	Receptor binding/ Fusion	Apoptosis	bcl-2	[111]
